# Preparation and Characterization of PVDF/PVPylated-TiO_2_ Composite Membrane with Enhanced Antifouling Performance

**DOI:** 10.3390/nano16020104

**Published:** 2026-01-13

**Authors:** Jie Zhang, Shiying Bo, Chunhua Wang, Qiancheng Xiong, Bingqiong Tan, Zicong Jian, Feiyan Xie, Jianpeng Li, Zicheng Xiao, Guocong Liu

**Affiliations:** 1 School of Chemistry and Materials Engineering, Huizhou University, 46 Yanda Road, Huizhou 516007, China; anglar@126.com (C.W.); xiong@hzu.edu.cn (Q.X.); bingqiongtan@163.com (B.T.); jackyken1984@gmail.com (Z.J.); xfy@hzu.edu.cn (F.X.); lijianpengljp@163.com (J.L.); zichxiao@hotmail.com (Z.X.); gcl_109@hzu.edu.cn (G.L.); 2 School of Environmental Science and Engineering, South University of Science and Technology of China, No. 1088 Xueyuan Avenue, Shenzhen 518055, China; 3 State Key Laboratory of Pollution Control and Resource Reuse, School of Environmental Science and Engineering, Tongji University, 1239 Siping Road, Shanghai 200092, China; bsytju@tongji.edu.cn

**Keywords:** PVPylated-TiO_2_, composite membrane, stability, hydrophilicity, antifouling performance

## Abstract

Hydrophilic modification of polymeric membranes by employing TiO_2_ nanoparticles has attracted much attention in enhancing antifouling performance. Micelles of PVPylated-TiO_2_ nanoparticles were designed to alleviate the agglomeration of TiO_2_ nanoparticles via steric hindrance and electrostatic stabilization effect. Herein, Poly(vinyl pyrrolidone) (PVP) was used as a surfactant to mitigate the thorny agglomeration of nanoparticles in the casting solution and simultaneously as a pore-forming additive during the membrane preparation process. The lowest backscattering (*BS*) peak and turbiscan stability index (*TSI*) of the composite casting solution indicated the effective dispersion and stabilization under the steric interaction of 4 wt.% PVP. Properties such as the fully developed finger-like structure of cross-sectional morphologies, water permeability, negative Zeta potential, and hydrophilicity were enhanced evidently by the optimal modification of PVPylated-TiO_2_ materials. High interaction energy indicated by classic extended Derjaguin–Landau–Verwey–Overbeek (XDLVO) theory as well as the high relative flux during the filtration of various model foulants demonstrated the effective antifouling modification. The results of critical flux and fouling rate in 30 min also verified the enhancement of the antifouling performance of PVDF/PVPylated-TiO_2_ composite membrane. This work provides a feasible strategy to construct composite membranes with high antifouling performance for wastewater treatment.

## 1. Introduction

In recent years, membrane technology is considered as one of the most promising separation technologies in the field of wastewater treatment due to its high efficiency, flexible operation, and energy saving characteristic [1,2]. Membrane material has been extensively applied in wastewater treatment due to the effective removal of particles, colloids, waterborne pathogens, and other pollutants [3]. However, membrane fouling, defined as the accumulation of foulants on the membrane surface or within its pores, is still the main obstacle in the application of membrane technology, reducing permeability and increasing energy consumption [4,5]. It is therefore important to develop strategies for membrane fouling control. One of the most fundamental and efficient strategies is fabricating antifouling membranes [6]. Hydrophilic modification is taken as one of the most promising methods to deal with the thorny fouling problem [7,8]. The antifouling performance is supposed to be promoted by introducing hydrophilic functional groups on membrane surfaces, which might enhance surface hydration and disable foulant adsorption via thermodynamic and steric fouling-resistant effects [9].

Organic materials and inorganic nanomaterials are normally adapted among numerous hydrophilic functional materials. Incorporation of hydrophilic polymers, such as poly (ethylene glycol) (PEG) or PVP, are commonly used to create a hydration layer hindering the interaction between foulants and the surface of the membrane, acting as both a physical and an energetic barrier deterring adsorption of the foulant [10]. In comparison with organic additives, inorganic materials are also generally used for the hydrophilic modification of membranes, owing to their strong chemical/thermal stability, high mechanical strength, and antibacterial ability [11]. TiO_2_ as a versatile material develops a bright application in membrane hydrophilic modification owing to its non-toxicity, thermal stability, biodegradability, high chemical stability, and good affinity to water [12,13]. TiO_2_ nanoparticles were designed to fabricate the composite membrane in view of imparting with the excellent characteristics in this study. PVDF was used as the basic polymeric membrane material in this study due to its outstanding properties, including high hydrophobicity, chemical and thermal stability, and excellent mechanical properties [14]. Blending hydrophilic TiO_2_ nanoparticles with PVDF as the simplest and most effective strategy was adopted to improve the antifouling performance in the study. Considering the drawback of the agglomeration of TiO_2_ nanoparticles induced by the high surface energy, the organic surfactant was taken into consideration. Hydrophilic organic polymer PVP with rich carbonyl functional groups was investigated as a pore-forming additive in membrane fabrication and a surfactant for TiO_2_ nanoparticle dispersion in this study [15,16]. TiO_2_ nanoparticles contain polar Ti-O bonds which make them have tremendous surface activity and can serve as adsorption carriers [17]. PVP could be adsorbed on the surfaces of TiO_2_ nanoparticles. Micelles of PVPylated-TiO_2_ nanoparticles were supposed to be formed. TiO_2_ nanoparticles adsorbed with PVP chains might help to alleviate the agglomeration of TiO_2_ nanoparticles via steric hindrance and the electrostatic stabilization effect. Therefore, to combine the advantages of both inorganic nanoparticles and organic material as well as the agglomeration mitigation of TiO_2_ nanoparticles, hydrophilic PVP was employed to improve the hydrophilicity of PVDF/TiO_2_ composite membranes.

The modification efficiency relies heavily on the dispersion of TiO_2_ nanoparticles, which may be closely related to the dosage of the surfactant, PVP. Casting solution stability might also correlate with the status of PVPylated-TiO_2_ nanoparticles, further impacting membrane property. A multiple light scattering spectroscopy (MLiSSP) was employed to explain the effect of various dosages of PVP on the stability of casting solutions. A transmission electron microscopy (TEM) was used to explore the agglomeration of TiO_2_ nanoparticles. The morphologies of PVPylated-TiO_2_ composite membranes were characterized by scanning electron microscope (SEM) and atomic force microscope (AFM). The physicochemical properties, including thickness, porosity, contact angle, and Zeta potential, were determined. The membrane antifouling performance was assessed by XDLVO theory. Filtration with various model foulants was conducted. Critical flux and fouling rate in an anoxic/oxic membrane bioreactor (A/O-MBR) were also determined to explore the antifouling performance in real wastewater treatment applications.

## 2. Materials and Methods

### 2.1. Materials and Experimental Set-Up

PVDF was purchased from Solvay Corporation (Brussels, Belgium). Dimethysulfoxide (DMSO) and dimethylacetamide (DMAC) used as solvents and PVP were obtained from Macklin (Shanghai, China). Anatase TiO_2_ nanoparticles with an average particle size of 21 nm was supplied by Sigma-Aldrich (St. Louis, MO, USA).

A pilot-scale submerged A/O MBR fed with raw municipal wastewater was set up in the Quyang municipal wastewater treatment plant (WWTP) of Shanghai. The schematic diagram of the device and the parameters of the design and operation conditions were illustrated in our previous study [18]. Bovine serum albumin (BSA, 67 kDa) which acted as a model protein foulant and sodium alginate which acted as a model polysaccharide foulant were purchased from Sinopharm (Shanghai, China). SMP (soluble microbial products), used as complex model foulants, was obtained by filtrating sludge in the aerobic zone of the A/O MBR device. The total organic carbon (TOC) in the SMP solution was measured by a TOC analyzer (TOC-VcPN, Shimadzu, Japan).

### 2.2. Membrane Preparation

PVDF/PVPylated-TiO_2_ composite membranes were prepared by phase inversion via the immersion precipitation method. Table 1 shows the composition of membranes. The components of the original PVDF membrane were set as 100% with 10 wt.% PVDF and 90 vol.% solvents. The contents of TiO_2_ nanoparticles and PVP were separately defined compared to the original polymeric casting solution. Casting solutions of the composite membranes were prepared by three steps. The first step was preparing homogenous polymeric solutions by dissolving 10 wt.% PVDF in partial solvent (about 30 vol.% DMSO and 30 vol.% DMAc) at 80 °C for 4 d. The second step was forming PVPylated-TiO_2_ solutions by dispersing TiO_2_ nanoparticles (0.15 wt.% in this study) with various contents of PVP (0 wt.%, 2 wt.%, 4 wt.%, and 6 wt.%) in the remaining solvents (about 15 vol.% DMSO and 15 vol.% DMAc) under ultrasonic agitation at 20 °C for 20 min. PVDF/PVPylated-TiO_2_ composite casting solutions were prepared in the last step by mixing various PVPylated-TiO_2_ solutions with homogenous polymeric solutions for 3 d at 80 °C. The casting solutions were subsequently casted on porous polyester non-woven fabrics and flat glass plates with a scraper clearance of 250 µm and a speed of 1.8 m/min. These casting films were then submerged into deionized water, which acted as a coagulation bath within 30 s at room temperature (about 25 °C). Membranes corresponding to various PVP content were named as P1, P2, P3, and P4. In addition, the morphology of various PVPylated-TiO_2_ nanoparticles in the second step was determined by a transmission electron microscopy (TEM, JEM 2011, JEOL Ltd., Tokyo, Japan).

### 2.3. Stability of Casting Solution

The stability of various casting solutions of PVPylated-TiO_2_ composite membranes was investigated by employing MliSSP (Turbiscan Tower, Formulaction, France). Two synchronous detectors, transmission (*T*) at an angle of 0°, and backscattering (*BS*) at an angle of 135° were employed to detect the movement of particles within casting solutions under a near-infrared light source (*λ* = 880 nm) at 80 °C for almost 16 h in this study [19]. Backscattering light signal (∆*BS*) and transmission (∆*T*) were analyzed along the height of the cylindrical glass within the samples from the bottom (0 mm) to the top (about 43 mm) with the original signals at 0 s as a reference [20]. A Turbiscan curve at a height of <3 mm of the glass container shows the sediment formed at the bottom, and the demixing thickness indicates the amount of sediment [21]. *TSI* is a statistical quantity calculated from the differences in the rate of backscattering or transmission intensity of the sample compared to the original by Equation (1) [22,23].

(1) $$TSI=\sqrt{\frac{\sum_{i=1}^{n}{\left(x_{i}-x_{BS}\right)}^{2}}{n-1}}$$

where Σ is the sum of all the scan differences from the bottom to the top of the tube, *x_i_* is the average backscattering for each minute of measurements, *x*_BS_ is the average *x*_i_, and *n* is the number of scans.

### 2.4. Membrane Characterization

The surface and cross-section morphologies of PVDF/PVPylated-TiO_2_ composite membranes P1–P4 were observed by SEM (FESEM, Hitachi S4800, Tokyo, Japan). The thickness of membranes with non-woven fabrics was measured by employing a micrometer at different areas. Porosity and water permeability of membranes were determined for three times, respectively, by employing the methods reported in our previous study [18]. Contact angle was determined by an optical contact angle measurement system (OCA 15 Plus, Data physics GmbH, Stuttgart, Germany). The zeta potential of membranes was measured by a streaming potential analyzer (EKA 1.00, Anton-Paar, Graz, Austria) with 10 mM KCl solution at a pH value of 7.0 as the flowing liquid. The attenuated total reflectance Fourier transform infrared (ATR-FTIR, Thermo scientific, Waltham, MA, USA) was adapted to analyze the functional groups on membrane surfaces over 4000–700 cm^−1^ at a resolution of 4 cm^−1^. The surface roughness parameters of membranes, characterized by average roughness (*R*a), root-mean-square roughness (*R*q), and maximum roughness (*R*max), were obtained by the analysis of three-dimensional morphologies of membrane surfaces, which were determined by AFM (Dimension 5000, Bruker AXS, Santa Barbara, CA, USA).

### 2.5. Antifouling Performance Assessment

#### 2.5.1. XDLVO Theory Analysis

XDLVO theory was employed to quantitively evaluate the adsorption of model foulants (i.e., SMP in this study) on membrane surfaces. The average size and zeta potential of SMP were measured by a Zetasizer analyzer (Nano-ZS90, Malvern Instruments, Malvern, UK). Surface tension parameters of membranes can be calculated by adapting the contact angles determined by applying three probe liquids (water, formamide, and diiodomethane) in extended Young’s Equations (2)–(4) [24,25,26].

(2) $$\gamma^{\mathrm{AB}}=2\sqrt{\gamma^{+}\gamma^{-}}$$

(3) $$\gamma^{\mathrm{TOT}}=\gamma^{\mathrm{LW}}+\gamma^{\mathrm{AB}}$$

(4) $$\left(1+\cos\theta \right)\;\gamma_{l}^{\mathrm{TOT}}=2\left(\sqrt{\gamma_{s}^{\mathrm{LW}}\gamma_{l}^{\mathrm{LW}}}+\sqrt{\gamma_{s}^{+}\gamma_{l}^{-}}+\sqrt{\gamma_{l}^{+}\gamma_{s}^{-}}\right)$$

where *γ^+^* means the electron acceptor parameter, *γ*^−^ means the electron donor parameter, and *θ* means the contact angle. *γ*^AB^, *γ*^LW^ and *γ*^TOT^ denote the AB component of surface tension, the LW component of surface tension, and the total surface tension, separately. The subscript (s) refers to either membrane surface or foulants (SMP in this study) and (*l*) refers to the probe liquid used in the measurements.

The free energy of adhesion (Δ*G_h_*_0_^TOT^) between membranes and SMP per unit area is the summation of Δ*G_h_*_0_^LW^, Δ*G_h_*_0_^AB^, and Δ*G_h_*_0_^EL^, which are Liftshiz–van der Waals (LW), acid–base (AB), and electrostatic (EL) interaction free energy components at the minimum separation distance *h*_0_ (*h*_0_ ≈ 0.158 nm) [27]. The corresponding components could be obtained by Equations (5)–(7), respectively. The free energy of cohesion for membranes can be obtained by replacing *γ*_m_ with *γ*_c_.

(5) $$\Delta G_{h_{0}}^{LW}=2\left(\sqrt{\gamma_{l}^{\mathrm{LW}}}-\sqrt{\gamma_{m}^{\mathrm{LW}}}\right)\left(\sqrt{\gamma_{c}^{\mathrm{LW}}}-\sqrt{\gamma_{l}^{\mathrm{LW}}}\right)$$

(6) $$\Delta G_{h_{0}}^{AB}=2\sqrt{\gamma_{l}^{+}}\left(\sqrt{\gamma_{m}^{-}}+\sqrt{\gamma_{c}^{-}}-\sqrt{\gamma_{l}^{-}}\right)+2\sqrt{\gamma_{l}^{-}}\left(\sqrt{\gamma_{m}^{+}}+\sqrt{\gamma_{c}^{+}}-\sqrt{\gamma_{l}^{+}}\right)-2\left(\sqrt{\gamma_{m}^{+}\gamma_{c}^{-}}+\sqrt{\gamma_{c}^{+}\gamma_{m}^{-}}\right)$$

(7) $$\Delta G_{h_{0}}^{\mathrm{EL}}=\frac{\kappa \varepsilon_{0}\varepsilon_{r}}{2}\left(\zeta_{c}^{2}+\zeta_{m}^{2}\right)\times \left(1-\coth(\right.\kappa h_{0})+\frac{2\zeta_{m}\zeta_{c}}{\left(\zeta_{c}^{2}+\zeta_{m}^{2}\right)}\csc h\left.\left(\kappa h_{0}\right)\right)$$

where *ε*_r_ denotes the dielectric constant of water, *ε*_0_ means the dielectric permittivity of vacuum, *ε*_0_*ε*_r_ means the dielectric permittivity of the fluid, and *ζ*_m_ and *ζ*_c_ denote the zeta potential of the membrane and SMP solution, respectively. The subscripts m, l, and c refer to membrane, bulk liquid (water in this study), and SMP in this study, respectively. The inverse Debye screening length, *κ*, can also be calculated [28]. The symbol and physical meaning of surface tension shown in Equations (5)–(7) are shown in Table 2.

The LW, AB, and EL interaction energy components between the membrane and SMP (*U*_mlc_^LW^, *U*_mlc_^AB^, and *U*_mlc_^EL^) can be obtained via Equations (8)–(10), respectively. The interaction energy between membrane surfaces and approaching foulants is the summation of above interaction energy components.

(8) $${U_{\mathrm{mlc}}}^{\mathrm{LW}}=2\pi \Delta G_{h_{0}}^{LW}\frac{h_{0}^{2}a}{h}$$

(9) $${U_{\mathrm{mlc}}}^{\mathrm{AB}}=2\pi a\lambda \Delta G_{h_{0}}^{AB}\exp\left[\frac{h_{0}-h}{h}\right]$$

(10) $${U_{\mathrm{mlc}}}^{\mathrm{EL}}=\pi \varepsilon_{0}\varepsilon_{r}a\left[2\zeta_{c}\zeta_{m}\ln\left(\frac{1+e^{-\kappa h}}{1-e^{-\kappa h}}\right)+\left(\zeta_{c}^{2}+\zeta_{m}^{2}\right)\ln(1-e^{-2\kappa h})\right]$$

where *a* is the radius of SMP, *h* is the separation distance between the membrane and SMP, and *λ* is the decay length of AB interactions (~0.6 nm).

#### 2.5.2. Foulant Filtration

A filtration experiment was conducted by applying a filtration cell (MSC300, Mosu Corporation, China) under a magnetic stirring rate of 500 rpm at room temperature to evaluate membrane antifouling properties. SMP, 1 g/L BSA, and 500 mg/L alginate sodium were adopted as model foulants. The concentration of foulants in the SMP solution was characterized by TOC. The membrane sample was pre-compressed by filtrating DI water for 30 min at 0.03 MPa and then conducting filtration by 250 mL of foulant solution. SMP was additionally filtrated by reverse osmosis (RO) membranes to obtain the SMP-fouled membrane for determining the contact angle of the SMP as documented in XDLVO theory analysis.

#### 2.5.3. Critical Flux and Fouling Rate

The critical flux of the membranes was determined by a stepwise method in the submerged A/O-MBR device. Flat-sheet modules of PVDF/PVPylated-TiO_2_ composite membranes P1–P4 with a filtration area of 0.38 m^2^ were prepared. The initial flux was set at a certain value, and the permeate flux was increased stepwise every 15 min with a flux step size of 2–3 L/(m^2^·h) without relaxation [29]. The critical flux was arbitrarily defined as the flux above which the increase in trans-membrane pressure (TMP) exceeded 0.4 kPa in 15 min. The critical flux was measured at approximately 22 °C in this study. The fouling rate of membranes named R30 in this study was measured under a constant flux of 60 L/(m^2^·h) in 30 min at approximately 22 °C to reflect the membrane fouling potentials. TMP was measured once every five minutes.

## 3. Results and Discussion

### 3.1. Stability Analysis of Casting Solution

**Figure 1:**
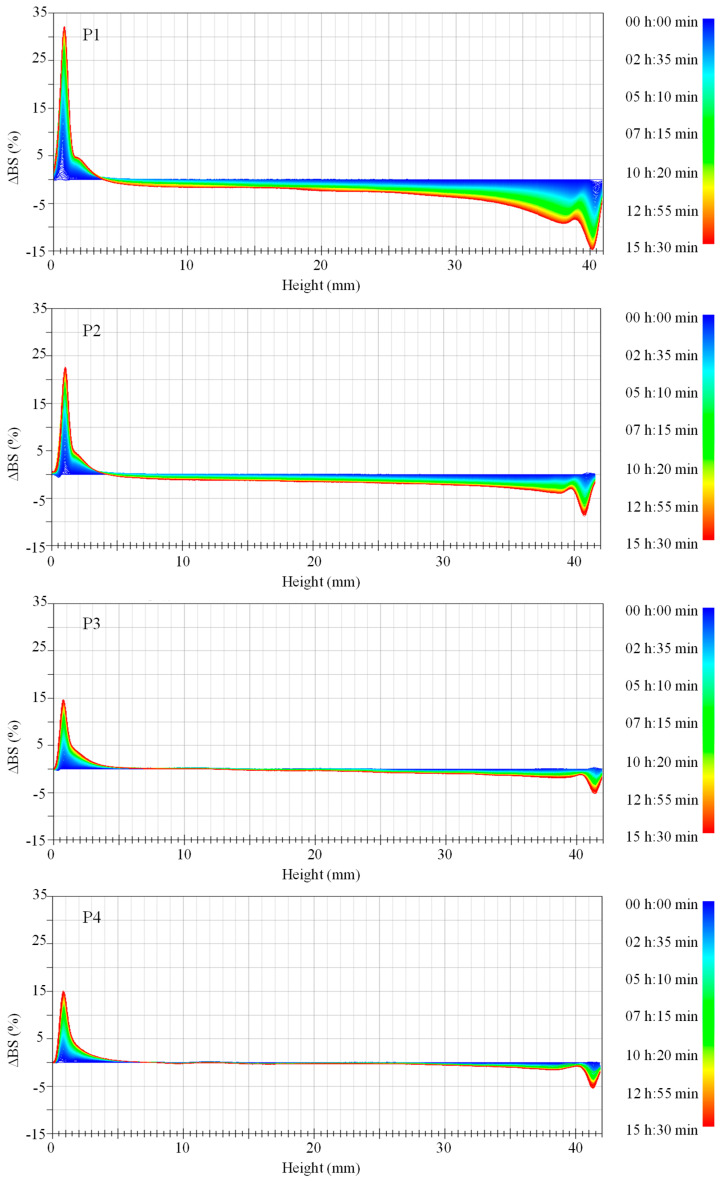
The backscattering intensity profiles along the sample height during the measuring period.

**Figure 2:**
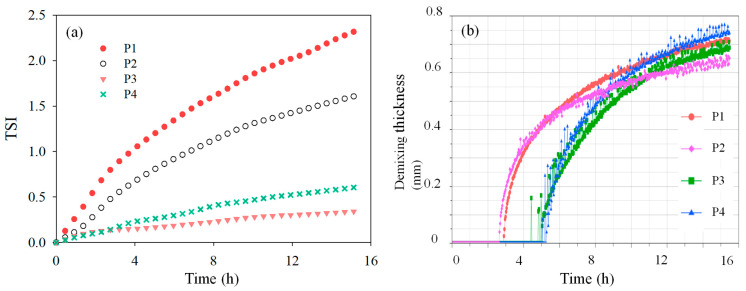
(**a**) *TSI* of casting solutions of PVDF/PVPylated-TiO_2_ composite membranes P1–P4 and (**b**) peak thickness at the bottom.

**Figure 3:**
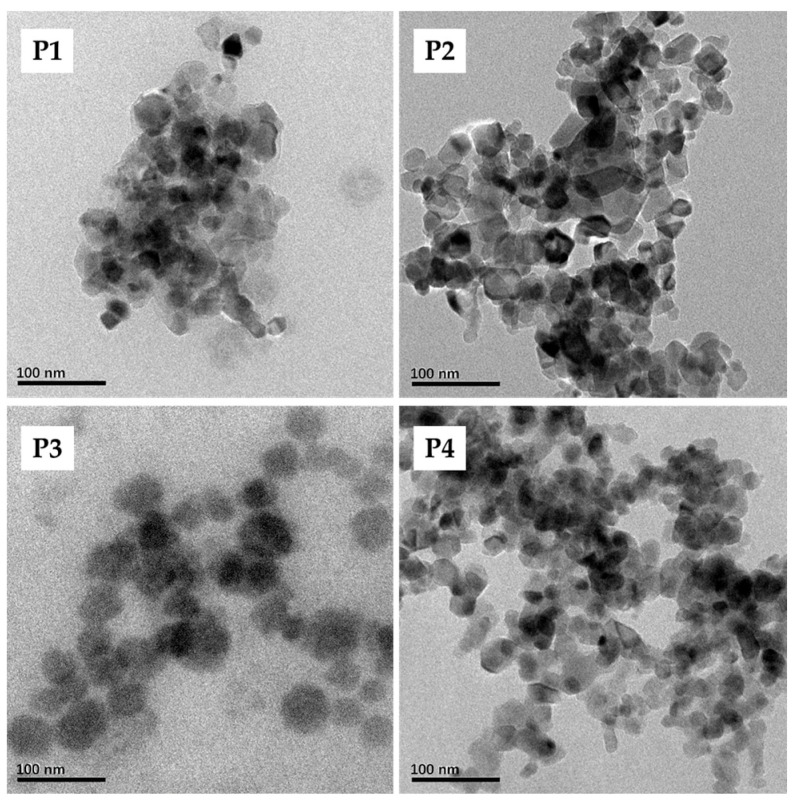
TEM images for various PVPylated-TiO_2_ nanoparticles.

The backscatter curves of the MLiSSP provide information of agglomeration, flocculation, and sedimentation of PVPylated-TiO_2_ nanoparticles in the casting solutions. As shown in Figure 1, the presence of the backscattering peak at a low height and the peak at a high height indicate the sedimentation process of PVPylated-TiO_2_ nanoparticles at the bottom and clarification at the top of casting solution, respectively [30]. The stability of the casting solution could be observed by the intensity of the peaks at both the bottom and the top. The highest peak at the bottom and the lowest peak at the top of the casting solution sample for neat PVDF/TiO_2_ composite membranes P1, shown in Figure 1, indicated the weakest stability. *TSI* is another important indicator to assess the stability of the casting solution. In addition, the smaller the value of *TSI*, the more stable the casting solution is [31]. The weakest stability of the casting solution for membrane P1 was also confirmed by the result of *TSI* displayed in Figure 2a. Both aggregation and sedimentation of nanoparticles could affect the dispersion stability of the casting solution [32]. Compared to neat TiO_2_ nanoparticles for membrane P1, PVPylated-TiO_2_ nanoparticles in the casting solutions for membrane P2–P4 exhibited the preferable dispersion, which was also demonstrated by the alleviative agglomeration of TiO_2_ nanoparticles under the steric interaction of surfactant PVP (shown in Figure 3). The lowest peak of ∆*BS* for the casting solution of PVDF/PVPylated-TiO_2_ composite membrane P3 (see Figure 1), the smallest *TSI* throughout the measurement time (see Figure 2a), and the weakest agglomeration of PVPylated-TiO_2_ nanoparticles (see Figure 3) verified the optimal dosage of surfactant PVP (4 wt.% in this study) for the formation of the well-dispersed PVPylated-TiO_2_ nanoparticles. Hydrophilic micelles formed by the hydrophilic interaction between the hydroxyl group on PVP and the TiO_2_ nanoparticles might contribute to the stable dispersion of the TiO_2_ nanoparticles in the casting solutions and their immobilization on membrane surfaces [33]. The peak thickness of the firm sediment for various casting solutions at the bottom, shown in Figure 2b, was about 0.70 ± 0.08 mm. The latter demixing process and the relative thin sedimentation peak for the casting solution of PVDF/PVPylated-TiO_2_ composite membrane P3 also demonstrated the excellent stability of the casting solution in conjunction with the preferable dispersion of TiO_2_ nanoparticles. Compared to other casting solutions, the entanglement of the long chains of PVP adsorbed on the surface of TiO_2_ nanoparticles might result in the earliest sedimentary phenomenon and the densest sediments in the casting solution of PVDF/PVPylated-TiO_2_ composite membrane P2. The long chains of the superabundant PVP adsorbed on the sedimentary PVPylated-TiO_2_ nanoparticles for composite membrane P4 were supposed to slightly increase the demixing thickness in view of the small burrs on the curve of peak thickness.

### 3.2. Membrane Characterizations

**Figure 4:**
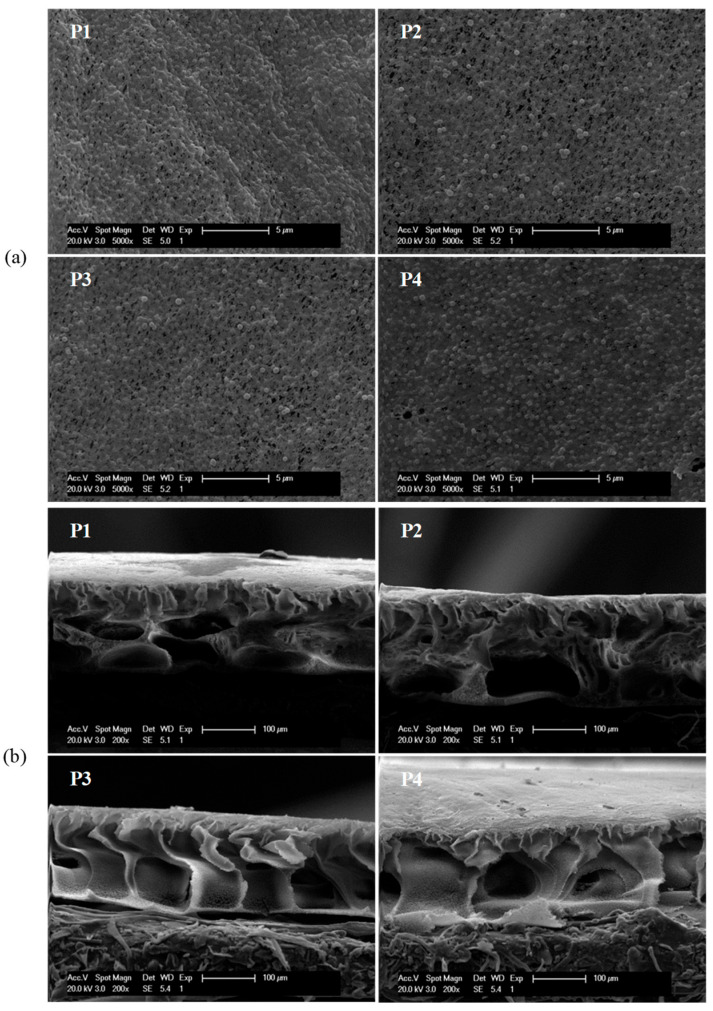
Morphologies of PVDF/PVPylated-TiO_2_ composite membranes P1–P4: (**a**) surface morphologies and (**b**) cross-sectional morphologies.

Surface structure was reported to greatly relate with the properties that govern protein adsorption and fouling resistance [8]. Surface structure was firstly monitored by SEM. Figure 4a shows the surface morphologies of PVDF/PVPylated-TiO_2_ composite membranes P1–P4. The modification of TiO_2_ nanoparticles with various PVP did not appreciably change the surface morphology of composite membranes. It can be observed from the cross-section morphologies (shown in Figure 4b) that all the membranes had the typical asymmetric structure of ultrafiltration membranes. Both PVDF/PVPylated-TiO_2_ composite membranes P1 and P2 have spongy-like structure with large connected macrovoids, while membranes P3 and P4 have fully developed finger-like structure on the cross-section morphology. The structure of the cross-section morphology is closely related to the exchange velocity between solvents and water during the phase inversion process [34]. The gradually developed macrovoids on the cross-section structures for membranes P1–P4 might be attributed to the accelerating exchange velocity induced by the high affinity to water of hydrophilic PVPylated-TiO_2_ nanoparticles with the increase in PVP dosages. The decreased thickness shown in Table 3 also verified the gradually enhanced exchange velocity during the phase inversion process in the coagulation bath. The closed, connected macrovoids observed on the cross-section morphology of membrane P4 might be attributed to a hindered exchange velocity, which perhaps resulted from the entanglement of luxuriant long chains of PVP between PVPylated-TiO_2_ nanoparticles. The porosity of PVDF/PVPylated-TiO_2_ composite membranes P1–P4 might be affected by the various cross-section structures. Table 3 shows relative high porosity and superior permeability for PVDF/PVPylated-TiO_2_ composite membrane T3, which is in accordance with the conjecture of membrane morphologies.

**Figure 5:**
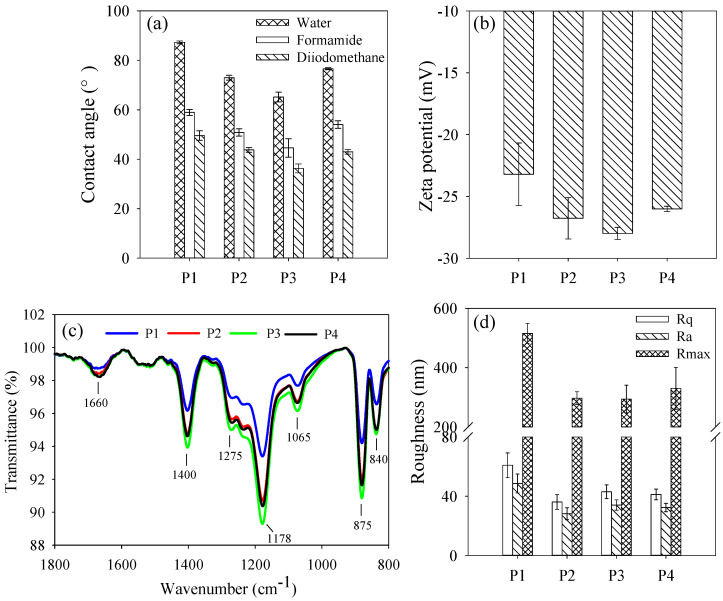
(**a**) Contact angles determined by employing three probe liquids, (**b**) zeta potentials, (**c**) ATR-FTIR spectra, and (**d**) roughness of PVDF/PVPylated-TiO_2_ composite membranes P1–P4.

The hydrophilicity of membranes is generally evaluated by contact angles, which are determined by employing water as the probe liquid [35]. As shown in Figure 5a, the contact angle of PVDF/PVPylated-TiO_2_ composite membrane P3 was obviously decreased by 25.3% compared to neat PVDF/TiO_2_ composite membrane P1. This might be due to the balanced distribution of hydrophilic PVP layers adsorbed on the surface of TiO_2_ nanoparticles [9]. Balanced nanospheres (PVPylated-TiO_2_ nanoparticles in this study) were supposed to be formed with TiO_2_ nanoparticles in the center adsorbed with PVP layers. The friendly matching structure might contribute to the even distribution on the surface of PVDF/PVPylated-TiO_2_ composite membrane P3, resulting in excellent hydrophilicity. With the further increase in PVP, PVPylated-TiO_2_ nanoparticles might be tangled on the cross-section structure, reducing the distribution on the membrane surface. This might also lead to the same tendency as the result of the zeta potential (see Figure 5b). Compared to neat PVDF/TiO_2_ composite membrane P1, the enhancement of negative zeta potential might be attributed to the carbonyl groups of PVP, which were sufficiently polarized to build up a negative charge density [36].

Figure 5c shows the ATR-FTIR spectra of various membranes. The full ATR-FTIR spectra are additionally shown in Figure S1. The peak at 1400 cm^−1^ was associated with the deformation vibration of -CH_2_. Peaks at 1275 cm^−1^ and 1178 cm^−1^ were taken as the symmetrical and asymmetrical stretching of -CF_2_. The peaks at 875 cm^−1^ and 840 cm^−1^ were, respectively, recognized as the characteristic peak of PVDF and the stretching vibration of -CH [33]. The peak at 1065 cm^−1^ was considered as the stretching vibration of -OH, derived from the hydrophilic TiO_2_ nanoparticles [28]. The peak at 1660 cm^−1^ was attributed to the adsorption of the carbonyl group in PVP chains [37]. The transmittance at the wavenumber confirmed the distribution of PVPylated-TiO_2_ nanoparticles on the surface of PVDF/PVPylated-TiO_2_ composite membranes P1–P4. The strong peak for membrane P3 demonstrated the optimal modification of hydrophilicity and the negative zeta potential. The appropriate distribution of hydrophilic micelles of PVPylated-TiO_2_ nanoparticles might benefit the hydration of water on the surface and enhancement of interfacial interaction energy, which might alleviate membrane fouling behavior [38]. Compared to neat PVDF/TiO_2_ composite membrane P1, the roughness of PVDF/PVPylated-TiO_2_ composite membranes P2–P4 (exhibited in Figure 5d) was probably decreased by the mitigated agglomeration of TiO_2_ nanoparticles, which might benefit from the formation of micelles of PVPylated-TiO_2_ nanoparticles. However, no obvious difference was found among the roughness of PVDF/PVPylated-TiO_2_ composite membranes P2–P4.

### 3.3. Assessment of Membrane Antifouling Performance

The interfacial interaction energies of various PVDF/PVPylated-TiO_2_ composite membranes were calculated using the XDLVO theory. Properties of SMP used as the model foulant in the theory calculation are displayed in Table S1. All the membranes exhibited relatively high electron donor components (*γ*^−^) and low electron acceptor components (*γ*^+^) (see Table 4) [39]. With the increase in PVP dosage under 4 wt.%, the electron donor component (*γ*^−^) increased gradually, indicating the enhancement of the electron donor monopolarity of membranes. This result might be due to the abundant hydrophilic groups present on PVPylated-TiO_2_ nanoparticles. However, *γ*^−^ of PVDF/PVPylated-TiO_2_ composite membrane P4 decreased otherwise with the further increase in PVP, which might be due to the deposition and immobilization of PVPylated-TiO_2_ nanoparticles on the cross-section structure and thus the reduced distribution on the surface. The free energies of cohesion per unit area point to the hydrophilic properties of the membrane material itself and the free energies of adhesion express the interaction tendency between membranes and foulants. A greater negative value implies a stronger attractive tendency for foulants, suggesting a weaker antifouling property [40]. As shown in Table 4, the free energy of adhesion between membrane surfaces and foulants (SMP in this study) shared the same tendency with the free energy of cohesion. With the formation of PVPylated-TiO_2_ nanoparticles for membranes P2–P4, the negative Δ*G*^TOT^ was improved compared to that of membrane P1. The high negative Δ*G*^TOT^ of both cohesion and adhesion for PVDF/PVPylated-TiO_2_ composite membrane P3 manifested an excellent antifouling property.

**Figure 6:**
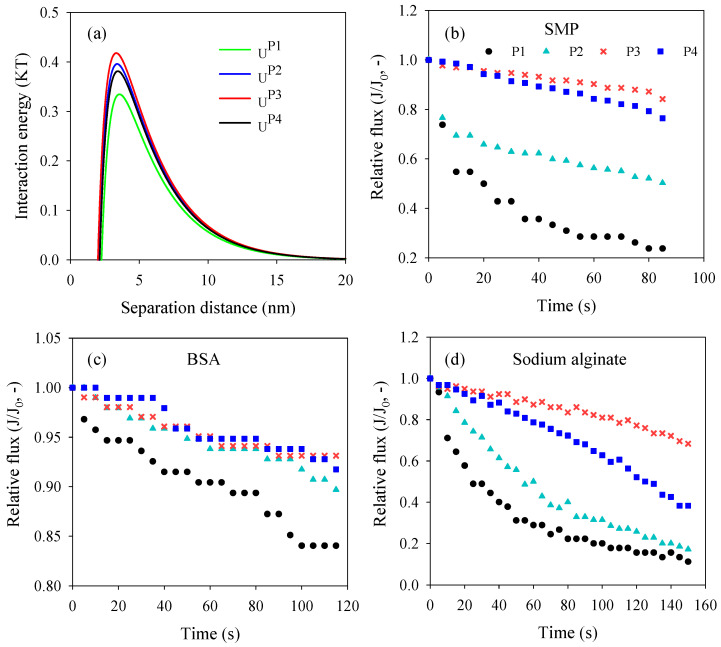
(**a**) Interaction energy between membrane surfaces and approaching foulants and normalized flux of membranes when filtrating (**b**) SMP solution, (**c**) BSA, and (**d**) sodium alginate solution.

Figure 6a shows the interaction energy between membrane surfaces and the approaching model foulants, SMP, along with separation distance. It is obvious that the interaction energy values are all positive at distances from membrane surfaces larger than about 2 nm, suggesting the repulsive interactions between the approaching SMP and membrane surfaces. The high interaction energy points to the high energy barrier for foulants to be attached on the membrane surface, implying the admirable antifouling performance. Therefore, PVDF/PVPylated-TiO_2_ composite membrane P3 exhibited the optimum antifouling property, which was confirmed by the high relative flux in the filtrating operation shown in Figure 6b. In order to further validate the antifouling modification, BSA and sodium alginate were additionally filtrated to explore the fouling process of protein and polysaccharide in this study (shown in Figure 6c,d). The high relative flux of PVDF/PVPylated-TiO_2_ composite membranes P2–P4 demonstrated the effective modification of PVP addition compared with the result of neat PVDF/TiO_2_ membrane P1. As shown in Figure 6c, the antifouling performance for protein was insensitive to the dosage of PVP above 4 wt.%. However, the antifouling performance for polysaccharide under 4 wt.% PVP (shown in Figure 6d) exhibited evident modification efficiency.

**Figure 7:**
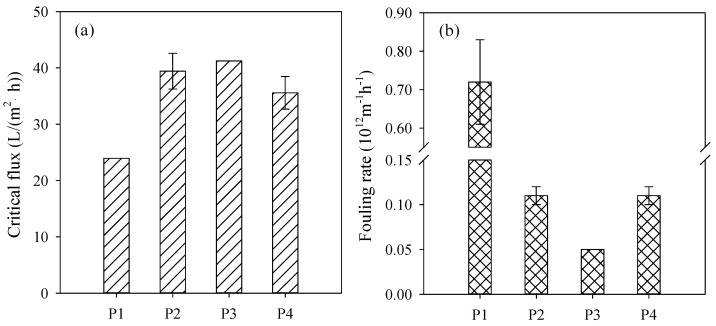
(**a**) Critical flux and (**b**) fouling rate in 30 min of PVDF/PVPylated-TiO_2_ composite membranes P1–P4 at 24 °C in A/O-MBR.

The critical flux and fouling rate measured under supra-critical flux are shown in Figure 7a,b, separately. These results indicated that PVDF/PVPylated-TiO_2_ composite membranes P2–P4 exhibited higher critical flux and lower fouling rate compared to the original PVDF/TiO_2_ membrane P1, manifesting the valid modification of PVP. Furthermore, the relatively high critical flux and low fouling rate for PVDF/PVPylated-TiO_2_ composite membrane P3 confirmed the optimal PVP dosage of 4 wt.%. This might be attributed to the enhanced hydrophilic property, negative zeta potential, and soft roughness surface, which resulted from the even formation of PVPylated-TiO_2_ nanoparticles as well as their distribution on the membrane surface. The decreased critical flux and fouling rate for PVDF/PVPylated-TiO_2_ composite membrane P4 further verified the optimal dosage of PVP.

## 4. Conclusions

PVDF/PVPylated-TiO_2_ composite membranes were prepared by employing various dosages of PVP. PVPylated-TiO_2_ nanoparticles were formed in advance for the purpose of mitigating agglomeration. Low peaks of ∆*BS* both at the bottom and top, *TSI* value, and later demixing time demonstrated the modified stabilities of casting solutions of PVP-modified membranes. The agglomeration of PVPylated-TiO_2_ nanoparticles was also improved to a different extent and the optimal dosage of PVP was testified to be 4 wt.%. With the PVPylated-TiO_2_ nanoparticles, macrovoids were fully developed into a finger-like structure on the cross-section morphology. Porosity and permeability, hydrophilicity, and the absolute value of the negative zeta potential were also obviously enhanced. The peaks at 1065 cm^−1^ and 1660 cm^−1^ confirmed the abundant distribution of hydrophilic and negatively charged PVPylated-TiO_2_ nanoparticles on membrane surfaces. The electron donor monopolarity, the free energy of cohesion and adhesion, as well as the interaction energy barrier between approaching soluble microbial products and membrane surfaces were also improved, suggesting the enhancement of antifouling ability. The high relative flux during the filtration of various foulants, as well as the high critical flux and low fouling rate, further verified the modification efficiency of PVPylated-TiO_2_ nanoparticles and the optimal dosage of PVP.

## Figures and Tables

**Table 1:** Detailed compositions of membranes P1–P4.

Membranes	PVDF (wt.%)	DMSO (vol.%)	DMAc (vol.%)	TiO_2_ (wt.%)	PVP (wt.%)
P1	10	45	45	0.15	0
P2	10	45	45	0.15	2
P3	10	45	45	0.15	4
P4	10	45	45	0.15	6

**Table 2:** Symbol and physical meaning of surface tension shown in Equations (5)–(7).

Symbol	Physical Meaning
$r_{m}^{LW}$	the LW component of surface tension for membrane
$r_{l}^{LW}$	the LW component of surface tension for probe liquid
$r_{c}^{LW}$	the LW component of surface tension for colloidal foulants
$r_{m}^{+}$	the electron acceptor parameter of surface tension for membrane
$r_{l}^{+}$	the electron acceptor parameter of surface tension for probe liquid
$r_{c}^{+}$	the electron acceptor parameter of surface tension for colloidal foulants
$r_{m}^{\_}$	the electron donor parameter of surface tension for membrane
$r_{l}^{\_}$	the electron donor parameter of surface tension for probe liquid
$r_{c}^{\_}$	the electron donor parameter of surface tension for colloidal foulants

**Table 3:** Properties of various PVDF/PVPylated-TiO_2_ composite membranes (n = 3).

Membrane No.	Thickness (mm)	Porosity (%)	Water Permeability (L/(m^2^·h·kPa)
P1	0.26 ± 0.01	38.1 ± 5.4	92.4 ± 9.6
P2	0.25 ± 0.01	47.2 ± 9.4	110.1 ± 3.3
P3	0.24 ± 0.01	50.9 ± 5.9	113.3 ± 6.0
P4	0.24 ± 0.00	48.4 ± 5.5	108.3 ± 2.3

**Table 4:** Surface tension parameters and surface free energy of PVDF/PVPylated-TiO_2_ composite membranes at the separation distance of *h*_0_ (*n* = 3).

		Surface Tension Parameters for Each Membrane (mJ/m^2^)								
Membrane	*γ* ^LW^	*γ* ^+^			*γ* ^−^		*γ* ^AB^			*γ* ^TOT^
P1	34.50 ± 1.05	0.73 ± 0.23			1.64 ± 0.51		2.12 ± 0.14			36.63 ± 0.92
P2	37.66 ± 0.49	0.53 ± 0.07			8.57 ± 0.19		4.24 ± 0.33			41.90 ± 0.81
P3	39.77 ± 0.84	0.36 ± 0.19			11.75 ± 0.50		3.98 ± 1.04			43.75 ± 1.86
P4	38.07 ± 0.50	0.32 ± 0.12			6.90 ± 0.95		2.90 ± 0.31			40.97 ± 0.80
	The Free Energy of Cohesion of Membranes (mJ/m^2^)						The Free Energy of Adhesion of Membranes (mJ/m^2^)			
Membrane	Δ*G*^LW^	Δ*G*^AB^	Δ*G*^EL^	Δ*G*^TOT^		Δ*G*^LW^		Δ*G*^AB^	Δ*G*^EL^	Δ*G*^TOT^
P1	−2.91 ± 0.43	−63.45 ± 1.70	0.19 ± 0.04	−66.17 ± 2.01		−3.12 ± 0.23		−64.73 ± 0.65	0.08 ± 0.01	−67.76 ± 0.85
P2	−4.31 ± 0.23	−36.75 ± 0.97	0.25 ± 0.03	−40.81 ± 0.71		−3.80 ± 0.10		−53.17 ± 0.65	0.10 ± 0.01	−56.87 ± 0.55
P3	−6.24 ± 0.47	−24.55 ± 1.50	0.27 ± 0.01	−30.52 ± 1.13		−4.57 ± 0.17		−48.43 ± 1.34	0.10 ± 0.00	−52.90 ± 1.17
P4	−4.51 ± 0.24	−43.55 ± 2.29	0.24 ± 0.00	−47.82 ± 2.52		−3.88 ± 0.10		−56.89 ± 0.53	0.09 ± 0.00	−60.68 ± 0.63

## Data Availability

The original contributions presented in this study are included in the article. Further inquiries can be directed to the corresponding author.

## Supplementary Materials

The following supporting information can be downloaded at https://www.mdpi.com/article/10.3390/nano16020104/s1; Figure S1: The full ATR-FTIR spectra of PVDF/PVPylated-TiO_2_ composite membranes P1–P4; Table S1: Properties of SMP (n = 3).
