# Supplementary material for: Preparation and Characterization of PVDF/PVPylated-TiO2 Composite Membrane with Enhanced Antifouling Performance
Source: Nanomaterials (Basel). 2026 Jan 13;16(2):104. doi: 10.3390/nano16020104 (PMC12844376; doi:10.3390/nano16020104)
Supplement: Supplementary file 1 [file nanomaterials-16-00104-s001.zip › nanomaterials-4056413-supplementary.pdf]

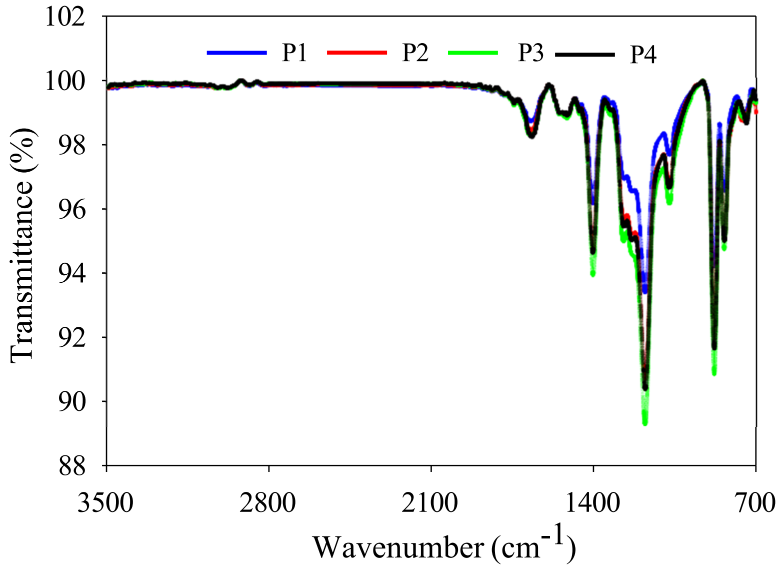

Figure S1. The full ATR-FTIR spectra of PVDF/PVPylated-TiO_2 composite membranes P1-P4.

Table S1. Properties of SMP (n=3).

TOC concentration (mg/L)	Average size (nm)	Zeta potential (mV)	Contact angle (°)		
			Water	Formamide	Diiodomethane
8.4±1.0	306.3±6.7	-10.4±0.4	95.7±2.1	65.3±3.0	47.7±0.8
